# Analysis of clinical risk factors for metabolic bone disease of prematurity

**DOI:** 10.3389/fped.2024.1345878

**Published:** 2024-05-16

**Authors:** Xiumin Liu, Ling Wang, Min Qian

**Affiliations:** ^1^ Department of Clinical Laboratory, The Second Hospital of Jilin University, Changchun City, Jilin Province, China; ^2^ Chongqing Yubei Center for Disease Control and Prevention, Chongqing, China; ^3^ Department of Neonatology, The Second Hospital of Jilin University, Changchun City, Jilin Province, China

**Keywords:** premature infant, metabolic bone disease, alkaline phosphatase, risk factors, newborn

## Abstract

**Objective:**

To analyze clinical data related to preterm infants and identify risk factors for metabolic bone disease of prematurity (MBDP).

**Methods:**

This study involved 856 newborns with a gestational age of less than 37 weeks or a weight of less than 1,500g at the Second Hospital of Jilin University. Multifactorial analysis was performed using logistic regression models to explore the risk factors for MBDP. Linear regression was used to investigate the factors affecting the time of alkaline phosphatase (ALP) exceedance and the peak value of ALP in the MBDP group.

**Results:**

In the MBDP group, ALP excesses occurred in preterm infants at an average of 39.33 days after birth, and the mean value of peak ALP was 691.41 IU/L. Parenteral nutrition and the application of assisted ventilation were independent risk factors for MBDP, with ORs of 1.02 and 1.03 respectively. Gestational age was found to be a protective factor for earlier time of onset of ALP exceedance (*β* = 2.24,) and the increase in the peak value of ALP (*β* = −16.30).

**Conclusion:**

Parenteral nutrition and the application of assisted ventilation are independent risk factors for MBDP. Gestational age is a major factor influencing the time of onset of ALP exceedance and the peak value of ALP in infants with MBDP.

## 1 Introduction

With the development of medical technology, the survival rate of preterm infants has been increasing in recent years, and the various complications of preterm infants are gradually receiving more attention. Metabolic bone disease of prematurity (MBDP) is a complication of preterm infants due to disorders in calcium and phosphorus metabolism in the body (1). The main clinical features of MBDP are elevated serum alkaline phosphatase (ALP) levels, hypophosphatemia, and skeletal hypomineralization (2, 3). If MBDP cannot be diagnosed and treated in time, the disease can lead to abnormal bone mineral content, decreased trabecular bone, other skeletal changes, and fractures, it can even lead to short stature and susceptibility to osteoporosis (4, 5).

In recent years, while the survival rate of preterm infants has improved in many developing countries, the recognition and prevention of MBDP in infants is still insufficient and needs urgent attention (6). The basic approach to MBD prevention involves improving calcium and phosphate intake from the first day of life, limiting the use of medications that increase bone resorption or calcium loss, promoting enteral feeding, and early identification of at-risk babies (7). As MBDP is usually asymptomatic in most infants, prevention and early diagnosis are the keys to the successful treatment of MBDP (8). ALP is a marker of bone turnover, and studies have found that ALP levels >500 IU/L were suggestive of impaired bone homeostasis and ALP levels >700 IU/L were associated with bone demineralization, despite the absence of clinical symptoms (9, 10). In addition, studies have shown that ALP levels >500 IU/L in infants were associated with MBDP (11).

MBDP is a multifactorial systemic disease that could be influenced by variety of conditions during intrauterine life and after birth (4). It has been found that preterm infants have lower lumbar spine bone mineral content and bone density than full-term infants of same age (12). The prevalence of MBDP has been estimated to be 23%–32% in low birth weight (<1,500g) infants and 50% in extremely low birth weight (<1,000g) infants (1, 13). Meanwhile, some treatments and interventions for preterm infants may also lead to the development of MBDP. Parenteral nutrition is a common risk factor for MBDP as the risk of calcium and phosphate precipitation limits the amount of these minerals in parenteral nutrition (6, 14). Also, some studies have indicated that patients with MBDP were more frequently associated with mechanical ventilation, chronic lung disease (11). Furthermore, maternal factors during the fetal period such as preeclampsia and intrauterine growth restriction (IUGR) have also been associated with an increased risk of MBDP in infants (2).

The main objective of this study is to identify the clinical risk factors of preterm infants at an early stage, which provides data support and factual basis for the diagnosis of MBDP. The secondary goal of the study is to expect a reduction in the incidence of MBDP or to mitigate the condition of MBDP.

## 2 Materials and methods

### 2.1 Study design and participants

This study was a retrospective analysis and was written according to the STROBE guidelines (15). Data were obtained from the Second Hospital of Jilin University, and the study population was 10,367 newborns which were born in the Department of Neonatology of the Second Hospital of Jilin University from November 2017 to December 2022. All surveys were reviewed and approved by the Medical Ethics Committee of the Second Hospital of Jilin University.

We selected newborns who met the following inclusion criteria: newborns with gestational age less than 37 weeks or weight less than 1,500g, and 942 newborns were included in the study. Meanwhile, newborns with no maternal medical record information or no ALP data were excluded. According to the specified inclusion and exclusion criteria, a total of 856 newborns were involved in the analysis.

### 2.2 Data extraction

Analytical data was extracted from hospital medical records of the Second Hospital of Jilin University. Record the following data: basic information of newborns, including gestational age, sex, birth weight, hospital stay, ALP, C-reactive protein (CRP), procalcitonin (PCT), direct bilirubin (DB), parenteral nutrition duration, assisted ventilation duration, and whether to apply glucocorticoid, sodium glycerophosphate; maternal information, including premature rupture of membranes (PROM), diabetes mellitus, severe preeclampsia, hypertension, history of birth asphyxia, IUGR and abnormal prenatal umbilical blood flow.

### 2.3 Relevant diagnostic criteria

In this study the research subjects were divided into two groups, ALP >500 IU/L was classified as MBDP group and ALP ≤500 IU/L as non-MBDP group (16). We also counted the time when ALP values started to exceed the standard and the peak value of ALP in the MBDP group. When CRP >5 mg/L or PCT >0.5 ng/ml, the neonate was considered to have an infection (17). DB >34 μmol/L was defined as cholestasis (18).

### 2.4 Bias

Bias is an unavoidable problem in epidemiological research. In this study, we used two individuals to collate the data individually to reduce the possibility of information bias in the data collection and collation process.

### 2.5 Statistical analysis

In this study, means and standard standard deviation (SD) were calculated to describe the continue variables. Meanwhile, categorical variables were described by the number of cases and percentage. One-way ANOVA and chi-square test were used for comparisons between groups. A multifactorial analysis was performed using logistic regression models to explore the risk factors for MBDP. Furthermore, linear regression was used to explore the factors influencing the time of onset of ALP exceedance and the peak value of ALP in the MBDP group. A two-sided *P* value <0.05 was considered a statistically significant difference.

## 3 Results

**Figure 1:**
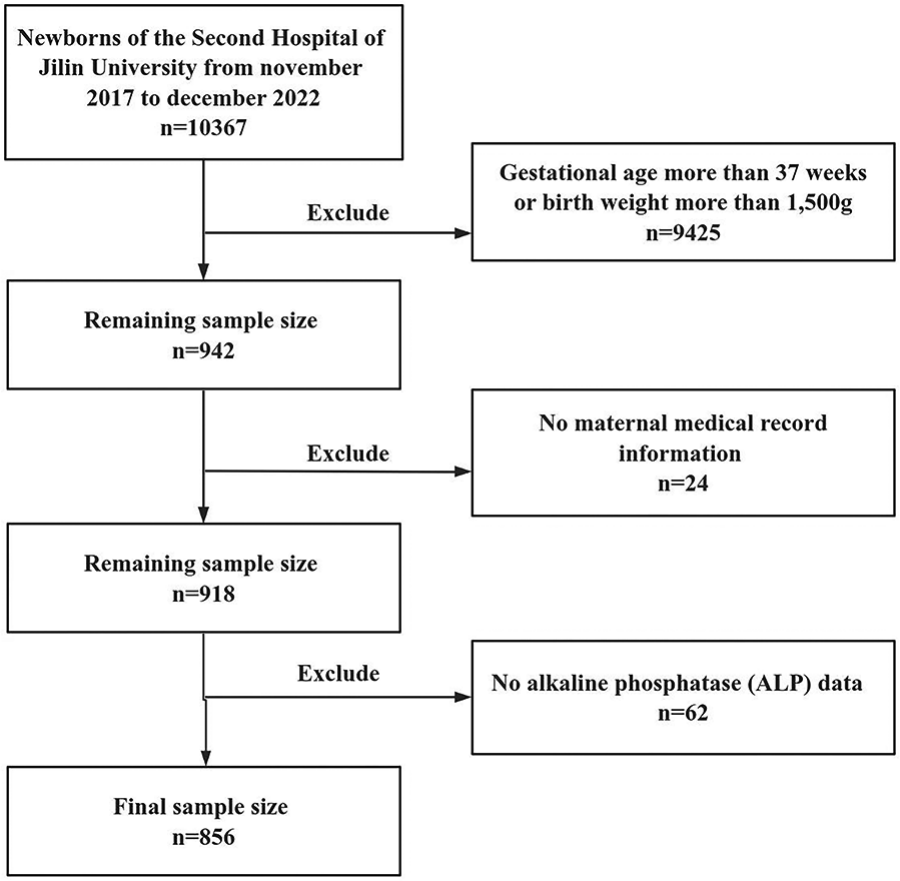
Flowchart of the sample.

A total of 856 participants were included in this study (Figure 1), with gestational age between 23.0 and 36.7 weeks and birth weight between 450 and 2,850g. Among them, there were 295 cases in the MBDP group and 561 cases in the non-MBDP group. Table 1 showed the basic information about the study population, and the general characteristics of the MBDP and non-MBDP groups. Compared with non-MBDP group, the MBDP group had lower gestational age and birth weight, higher parenteral nutrition duration and assisted ventilation duration, as well as longer hospital stay (*P* < 0.001). Moreover, the MBDP group had higher use of glucocorticoid and sodium glycerophosphate use, and higher proportion of cholestasis than the non-MBDP group (*P* < 0.001). In the MBDP group, ALP excesses occurred in preterm infants at a mean of 39.33 days after birth and the mean value of peak ALP was 691.41 IU/L.

**Table 1:** Basic conditions of the studied preterm infants.

Variables Mean (SD)/*n* (%)	Total	MBDP		*F*/*χ*^2^	*P*
	*N* = 856	No (*N* = 561)	Yes (*N* = 295)		
Gestational age, weeks	29.87 (2.31)	30.1 (2.47)	29.46 (1.94)	14.64	<0.001
Sex				3.771	0.052
Male	428 (50.0)	267 (47.6)	161 (54.6)		
Female	428 (50.0)	294 (52.4)	134 (45.4)		
Birth weight, g	1,223.8 (324.95)	1,264.83 (342.37)	1,145.66 (272.81)	26.71	<0.001
Parenteral nutrition duration, days	41.18 (26.46)	33.57 (20.4)	54.26 (30.32)	117.06	<0.001
Assisted ventilation duration, days	55.35 (25.65)	47.5 (23.75)	68.88 (23.07)	136.18	<0.001
Glucocorticoid				15.958	<0.001
Unused	124 (17.5)	98 (21.8)	26 (10.0)		
Used	585 (82.5)	351 (78.2)	234 (90.0)		
Sodium glycerophosphate				12.691	<0.001
Unused	449 (52.5)	319 (56.9)	130 (44.1)		
Used	407 (47.5)	242 (43.1)	165 (55.9)		
Infection				0.522	0.470
No	539 (66.5)	352 (67.4)	187 (64.9)		
Yes	271 (33.5)	170 (32.6)	101 (35.1)		
Cholestasis				14.535	<0.001
No	776 (90.6)	524 (93.4)	252 (85.4)		
Yes	80 (9.4)	37 (6.6)	43 (14.6)		
hospital stay, days	61.45 (31.38)	52.81 (27.29)	77.88 (32.13)	144.05	<0.001
Time of onset of ALP exceedance	–	–	39.33 (25.87)		
Peak value of ALP	–	–	691.41 (189.36)		

Table 2 presented the characteristics of the maternal gestation period. As shown in the table, the percentage of hypertension in the MBDP group was lower than that in the non-MBDP group (*P* < 0.05). The differences in PROM, diabetes mellitus, severe preeclampsia, history of birth asphyxia, IUGR and abnormal prenatal umbilical blood flow were not statistically significant between the MBDP and non-MBDP groups (*P* > 0.05).

**Table 2:** Characteristics of the maternal gestation period.

Variables *n* (%)	Total	MBDP		*χ* ^2^	*P*
	*N* = 856	No (*N* = 561)	Yes (*N* = 295)		
PROM				0.035	0.851
No	607 (70.9)	399 (71.1)	208 (70.5)		
Yes	249 (29.1)	162 (28.9)	87 (29.5)		
Diabetes mellitus				0.254	0.615
No	650 (75.9)	423 (75.4)	227 (76.9)		
Yes	206 (24.1)	138 (24.6)	68 (23.1)		
Severe preeclampsia				0.826	0.363
No	625 (73.0)	404 (72.0)	221 (74.9)		
Yes	231 (27.0)	157 (28.0)	74 (25.1)		
Hypertension				4.541	0.033
No	518 (60.5)	325 (57.9)	193 (65.4)		
Yes	338 (39.5)	236 (42.1)	102 (34.6)		
History of birth asphyxia				2.021	0.568
No	706 (82.5)	463 (82.5)	243 (82.4)		
Mild	60 (7.0)	39 (7.00)	21 (7.1)		
Moderate	61 (7.1)	37 (6.6)	24 (8.1)		
Severe	29 (3.4)	22 (3.9)	7 (2.4)		
IUGR				2.171	0.141
No	839 (98.0)	547 (97.5)	292 (99.0)		
Yes	17 (2.0)	14 (2.5)	3 (1.0)		
Abnormal prenatal umbilical blood flow				0.072	0.789
No	837 (97.8)	548 (97.7)	289 (98.0)		
Yes	19 (2.2)	13 (2.3)	6 (2.0)		

The relevant factors selected from the above univariate analysis were subjected for multivariate logistic regression analysis (Table 3). The results showed that both the parenteral nutrition duration and the assisted ventilation duration were influential factors for MBDP, with ORs of 1.02 (*P* < 0.001) and 1.03 (*P* < 0.001), respectively.

**Table 3:** Logistic regression analysis of MBDP influencing factors.

Variables	OR	95% CI	*P*
Parenteral nutrition duration, days	1.02	1.01, 1.03	<0.001
Assisted ventilation duration, days	1.03	1.02, 1.04	<0.001

In addition, we used linear regression models to analyze the factors influencing the time of onset of ALP exceedance in the MBDP group (Table 4). The results of univariate analysis showed that gestational age, birth weight, parenteral nutrition duration, assisted ventilation duration, sodium glycerophosphate and PROM all had a statistically significant effect on the time of onset of ALP exceedance in the MBDP group (*P* > 0.05). Further multifactorial analyses revealed that for each unit increase in gestational age, the time of onset of ALP exceedance would be 2.24 units later. In addition, an increase in the parenteral nutrition duration (*β* = 0.32, *P* < 0.001) and assisted ventilation duration (*β* = 0.27, *P* = 0.004) also slightly delayed the time of onset of ALP exceedance.

**Table 4:** Linear regression analysis of factors affecting the time of onset of ALP exceedance in MBDP group.

Variables	*β*	SE	*P*
Single-factor analysis			
Gestational age	−1.96	0.77	0.012
Birth weight	−0.02	0.01	<0.001
Parenteral nutrition duration, days	0.41	0.05	<0.001
Assisted ventilation duration, days	0.46	0.07	<0.001
Sodium glycerophosphate	6.05	3.02	0.046
PROM	−7.35	3.28	0.026
Multi-factor analysis			
Gestational age	2.24	0.91	0.015
Parenteral nutrition duration, days	0.32	0.06	<0.001
Assisted ventilation duration, days	0.27	0.09	0.004

Meanwhile, Table 5 showed the analysis of factors affecting the peak value of ALP in the MBDP group. The results of univariate analysis showed that gestational age, birth weight, parenteral nutrition duration, had a statistically significant effect on the peak value of ALP in the MBDP group (*P* > 0.05). Multifactorial analysis revealed that gestational age was a protective factor for the increase in the peak value of ALP (*β* = −16.30, *P* < 0.001).

**Table 5:** Linear regression analysis of factors affecting the peak value of ALP in MBDP group.

Variables	*β*	SE	*P*
Single-factor analysis			
Gestational age	−15.11	5.68	0.008
Birth weight	0.92	0.40	0.021
Parenteral nutrition duration, days	1.14	0.52	0.030
Multi-factor analysis			
Gestational age	−16.30	6.47	0.012

## 4 Discussion

In this study, we analyzed the correlation between clinical factors and the occurrence of MBDP, and found that the application of parenteral nutrition and assisted ventilation were independent risk factors for MBDP. In addition, gestational age was the main factor influencing the time of onset of ALP exceedance and the peak value of ALP in infants with MBDP, the parenteral nutrition duration and assisted ventilation duration also had a certain effect on the time of onset of ALP exceedance.

It has been reported that MBDP occurs in 16%–40% of extremely low birth weight newborns (4, 11). In our study population, the total prevalence of MBDP in infants with gestational age <37 weeks or birth weight <1,500g was 34.5% (295/856), which was generally consistent with previous reports. During fetal life, bone mineral accretion is maximal in the third trimester, and studies have shown that the fetus will obtain nearly 80% of its calcium and phosphorus reserves in the last 3 months of pregnancy (14, 19). For preterm infants, each day's reduction in gestational age has a huge impact on their bone growth (20). A meta analysis indicates that the smaller the gestational age, the higher the risk of MBDP (21). Although gestational age was not an independent risk factor for MBDP in our study, the results showed that the smaller the gestational age, the earlier the time of onset of ALP exceedance and the higher the peak value of ALP in the MBDP group. Prematurity makes it difficult for newborns to maintain an equivalent mineral intake after birth, thus affecting the process of neonatal bone mineralization (19, 22), it might exacerbate the rate of increase in ALP values. Studies have shown that neonatal serum ALP is 90% of bone origin and the gestational week plays a critical role in ALP activity in preterm and term infants, implicating that preterm infants are in high risk for bone metabolic diseases and have higher ALP values (23).

Our study found that the longer parenteral nutrition duration was a risk factor for MBDP. For physiological reasons, preterm infants often have to use parenteral nutrition after birth, however, parenteral nutrition usually does not contain enough calcium and phosphorus to fully meet the needs of preterm infants for bone mineralization (24). Therefore, preterm infants might need to be supplemented with other nutrients, which could lead to aluminum contamination. One study reported that preterm infants receiving parenteral nutrition for >3 weeks had 10-fold higher levels of bone aluminum than control group (25). Some studies have indicated that aluminum-contaminated parenteral nutrition may also contribute to MBDP (26). Notably, an increase in the parenteral nutrition duration in our study may have slightly delayed the time of onset of ALP exceedance in the MBDP group. It has been suggested that ALP levels are also affected by the timing of the application of parenteral nutrition (27). It suggests that in the future use of parenteral nutrition for preterm infants, we should focus not only on duration, but also on the timing of the start of application as well as other aspects of details. Meanwhile, a Japanese study found that when preterm infants were given a high-phosphorus intake by parenteral nutritional, it guaranteed a high calcium intake, leading to a decrease in ALP levels in the first month (28). We suspect that the greater amount and better absorption of calcium and phosphorus in intravenous nutrition compared to oral supplementation might attenuate or delay the occurrence of MBDP.

Bronchopulmonary dysplasia (BPD) is a common cause of respiratory illness in preterm newborns, and preterm infants with BPD often require more ventilation support (29). And some studies have found that BPD and the duration of noninvasive ventilation were associated with the development of MBDP (7). Similarly, in our study, assisted ventilation was an independent risk factor for MBDP. In preterm infants, the increasing prevalence of BPD, with a concomitant prolonged duration of assisted ventilation (30, 31). Research has demonstrated that newborns require immobilization during assisted ventilation, and the prolonged periods of immobility result in reduced motor stimulation which can potentially lead to bone mineralization defects (32). In our study, prolonged duration of assisted ventilation was associated with delayed time of onset of ALP exceedance in the MBDP group. It has been suggested that the relationship between MBDP and assisted ventilation was probably influenced by exercise, and that passive movement of newborn's limbs could potentially enhance bone mineralization even with the application of assisted ventilation (33). During the application of assisted ventilation, it is recommended to ensure basic activities for newborns without requiring additional exercises (34). On the other hand, longer duration of assisted ventilation usually implies poorer lung function in newborns, and in our study, MBDP appeared later in those newborns. Previous studies have usually considered the relationship between neonatal lung disease and MBDP (7, 30, 35), and have lacked the exploration of the time of onset, the results of this study may provide some reference.

In our univariate analysis, several other factors such as birth weight, glucocorticoids, sodium glycerophosphate, cholestasis, and hypertension were also found to have statistically significant differences between the MBDP and non-MBDP groups. These factors have also been shown to be associated with the development of MBDP in other studies (21, 36–38). Our study comprehensively considered prenatal and postnatal factors in newborns and provides certain theoretical support for future research on the prevention, screening and treatment of MBDP. In this study, preterm birth, parenteral nutrition and assisted ventilation were the most significant risk factors for MBDP, further research is needed to optimize nutritional practices in early life and to better manage the treatment of assisted ventilation, and most importantly to focus on the management of maternal health to prevent prematurity.

Our study also has some limitations. Firstly, our study is a retrospective study, which the reliability of causal conclusions may not be as robust. Secondly, due to the limited number of detection items in the sample, the MBDP judgment criteria were based on ALP values only, and the influencing factors obtained from the results may not be comprehensive. Thirdly, the population surveyed in this study was small and the results cannot yet be extrapolated, and further research is necessary to explore the exact association among them.

## 5 Conclusions

In conclusion, parenteral nutrition and the application of assisted ventilation are independent risk factors for MBDP; meanwhile, gestational age is a major factor influencing the time of onset of ALP exceedance and the peak value of ALP in infants with MBDP. Therefore, maternal health care should be strengthened to address the corresponding risk factors, reduce preterm delivery, start enteral nutrition as early as possible, and shorten the duration of assisted ventilation in order to reduce the occurrence of MBDP.

## Data Availability

The raw data supporting the conclusions of this article will be made available by the authors, without undue reservation.
